# C-855-03. Burn Injury Type Influences Arrhythmia Outcomes with Implications for Post-burn Cardiac Monitoring

**DOI:** 10.1093/jbcr/irag033.087

**Published:** 2026-04-06

**Authors:** Snehal A Kumar, Victoria Teoh, Amina El Ayadi, Steven E Wolf, Juquan Song

**Affiliations:** University of Texas Medical Branch, John Sealy School of Medicine, Galveston, TX; University of Texas Medical Branch, Galveston, TX; Division of Burn and Trauma Surgery, Department of Surgery, University of Texas Medical Branch, Galveston, Galveston, TX; Division of Burn and Trauma Surgery, Department of Surgery, University of Texas Medical Branch, Galveston, Galveston, TX; University of Texas Medical Branch, Galveston, TX

## Abstract

**Introduction:**

Hypovolemic shock is a common complication of burns involving more than 30% of total body surface area, creating substantial cardiac stress to maintain organ perfusion. Electrical burns occur through direct current transfer to an individual’s body during events such as circuit completion due to tissue conductivity, contact with faulty power lines, or lightning strikes. Current knowledge on the effects of burn-type on cardiac function is limited. This study investigates the effects of electrical, chemical, and thermal burns on the cardiac condition system, with particular emphasis on arrhythmias.

**Methods:**

A retrospective cohort study was conducted using TriNetX, a real-world health data platform. Adult patients with burn injuries diagnosed pre-2023 across 71 U.S. healthcare organizations were grouped based on the type of burn injury sustained: electrical, thermal, or chemical. Patients with hereditary channelopathies, congenital heart block, certain autoimmune conditions, history of thyrotoxicosis, and prior use of antiarrhythmics were excluded. 1:1 propensity-score matching (PSM) was performed across 21 covariates, including demographics and comorbid diseases. Outcomes pertaining to abnormal heart rhythms over 1 and 2 years post-burn injury were evaluated using risk ratios (RR), hazard ratios (HR), and Kaplan–Meier analysis. 95% confidence intervals (CI) and significant *p*-values (< 0.05) were reported.

**Results:**

After PSM, cohort sizes varied from 5119 to 131 006 patients per group. Electrical burns were associated with higher risk of premature ventricular contractions (PVC) 2 years post-injury compared to thermal burns (RR 2.29, CI 1.22-4.29). Risk of bundle branch block (BBB) was elevated in patients with electrical burns compared to chemical burns 2 years post-injury (RR 1.86, CI 1.05-3.29). Thermal burns were linked to greater risk of supraventricular tachycardia (SVT) 1 and 2 years post-injury only when compared to chemical burns (1 year: RR 1.22, CI 1.02-1.44; 2 years: RR 1.14, CI 1.00-1.31). Kaplan–Meier analyses supported these results.

**Conclusions:**

Direct electrical shock can dysregulate the heart’s conduction system, damaging myocyte gap junctions. In this study, electrical burns were associated with elevated risk of PVCs and BBBs compared to thermal and chemical burns, respectively. Thermal burns increased the risk of SVT, potentially due to electrolyte imbalances triggered by fluid loss. These findings highlight the distinct arrhythmia outcomes across burn types, and emphasize the need for further investigation of specific mechanisms related to cardiac conduction system disruption post-burn.

**Applicability of Research to Practice:**

Patients might benefit from proactive measures and arrhythmia-specific cardiac monitoring depending on burn type.

**Funding for the study:**

N/A.

